# An in vitro study on the antimicrobial efficacy of a calcium hydroxide versus a calcium silicate-based endodontic medicament

**DOI:** 10.1007/s00784-025-06524-w

**Published:** 2025-10-14

**Authors:** Dheepthi Jana, Eda Dzinovic, Ahmed Almaroof, Dipti Mehta, Sherif Elsharkawy, Sanjukta Deb, Sadia Niazi

**Affiliations:** 1 Department of Endodontics, Faculty of Dentistry, Oral & Craniofacial Sciences, King’s College London, London, SE1 9RT UK; 2 Centre for Oral, Clinical & Translational Sciences, Faculty of Dentistry, Oral & Craniofacial Sciences, King’s College London, SE1 9RT London, UK; 3 Department of Conservative Dentistry, College of Dentistry, University of Baghdad, 10071 Baghdad, Iraq

**Keywords:** Calcium hydroxide, Antimicrobial activity, Cell viability, Alkalinity, Intracanal medicaments, Calcium silicate, Endodontic treatment, Dental materials

## Abstract

**Background and objectives:**

Intracanal medicaments are essential adjuncts to chemo-mechanical canal preparation when controlling endodontic infections; however, their antibacterial action may not be uniform due to the diversity of the involved species. This study aimed to compare the long-term antimicrobial efficacy, metabolic activity, and pH of two intracanal medicaments: calcium silicate-based BC Temp (BC) and DEHP calcium hydroxide (CH).

**Materials and methods:**

The pH was examined over a 28-day period with and without freshly extracted tooth-root sections. Antimicrobial activity was assessed using a direct contact test (DCT) against *Enterococcus faecalis*, *Streptococcus mitis/oralis*, *Cutibacterium acnes*, and *Staphylococcus epidermidis*, by quantifying colony-forming units (CFU). The metabolic activity and cell viability were measured using the colorimetric 2,3-Bis-(2-methoxy-4-nitro-5-sulfophenyl)-2H-tetrazolium-5-Carboxanilide (XTT) assay. Statistical analysis was conducted using a two-way analysis of variance (ANOVA) followed by a post-hoc Tukey's and Šídák's tests.

**Results:**

Both CH and BC demonstrated high alkalinity (pH 11–13), with CH maintaining higher pH for a longer period (14 days) compared to BC (7 days). No significant differences were observed when the pH of HBSS was measured with or without a sectioned tooth-root surface in contact with tested medicaments. Both medicaments exhibited effective antimicrobial activity against the tested planktonic bacteria. CH demonstrated rapid and superior antibacterial efficacy against *E. faecalis and S.epidermidis* at 48 h, while BC, which has a smaller particle size, sustained CFU reduction at later time points (21 and 28 days), particularly against *S.mitis/oralis, S.epidermidis, and E.faecalis*. Furthermore, the XTT assay indicated reduced cell viability following treatment with both CH and BC, with BC demonstrating a more stable effect over time and maintaining cell viability below 50% across all bacterial groups during 28-day period.

**Conclusions:**

While maintaining a pH similar to that of CH, which has a well-established antibacterial effect; the calcium silicate-based BC Temp exhibited enhanced antimicrobial activity against the tested bacteria.

## Introduction

Microorganisms and their products are widely regarded as the primary aetiological factor of endodontic disease [1]. Studies have reported the existence of bacterial colonies within infected root canals, predominantly comprising anaerobic bacteria, which account for 80% to 93% of the total bacterial population [2, 3]. In addition to the root canal, these microorganisms also infiltrate hard-to-reach areas of dentine, including dentinal tubules, fins, accessory canals, ramifications, apical deltas, and transverse anastomoses [4]. These anatomical complexities provide the bacteria protection from immune cells, making complete removal via chemo-mechanical preparation alone challenging, thus increasing the risk of reinfection [5, 6]. Consequently, intracanal medicaments are frequently utilised to complement chemo-mechanical preparation in eradicating remaining microbes [7].

Calcium hydroxide (Ca(OH)_2_) has traditionally been the preferred intracanal medicament for primary endodontic lesions due to its antimicrobial properties, ability to dissolve tissue, and deposit mineralised tissue [7–9]. However, studies have demonstrated the material’s failure to eliminate microorganisms present in secondary endodontic infections, particularly *Enterococcus faecalis* [10]. Furthermore, prolonged use of calcium hydroxide may increase the risk of root fracture [11].

The application of calcium silicate-based bioceramics for treatment of endodontic disease has been growing due to their excellent bioactivity and biocompatibility. The material exerts its desired effects by a bi-phase of the setting reaction; hydration when in contact with water which causes the release of calcium hydroxide, followed by the precipitation phase when in contact with calcium phosphate in tissue fluids, resulting in the formation of hydroxyapatite [12].

In this study, we conducted an in-vitro assessment of the antimicrobial activity of a calcium silicate-based medicament, BC Temp (BC). According to the manufacturer, BC is clinically indicated in pulp necrosis, root canal retreatment, perforation management, apexogenesis, and treatment of internal and external resorption prior to the use of a bioceramic putty. The material has a high alkalinity (pH of 12 ± 1) and high radiopacity (> 9 mm Al) that complies with ISO 6876.

The antimicrobial properties of both BC and calcium hydroxide DEHP (CH), a commonly used intracanal medicament, were tested against planktonic microbes, Gram-positive bacteria, including *Enterococcus faecalis* (*E. faecalis*), *Streptococcus mitis/oralis* (*S. mitis/oralis*), *Cutibacterium acnes* (*C. acnes*), and *Staphylococcus epidermidis* (*S. Epidermidis*) to evaluate their antimicrobial activity. Furthermore, the metabolic activity and viability of biofilms were measured using the colorimetric 2,3-Bis-(2-Methoxy-4-Nitro-5-Sulfophenyl)−2H-Tetrazolium-5-Carboxanilide (XTT) assay, while the pH values of the medicaments were monitored over a 28-day period.

## Material and methods

The intracanal medicaments, their manufacturers and chemical compositions are outlined in Table 1.

**Table 1:** The chemical composition and manufacturers of intracanal medicaments tested in this study

Medicament	Manufacturer	Availability	Composition
DEHP (Calcium hydroxide CH)	DE Healthcare Ltd, Northampton, UK	Preloaded syringe	Calcium hydroxide, barium sulfate, and Polypropylene glycol
BC Temp (Bioceramic BC)	Brasseler, Savannah, USA	Preloaded syringe	Calcium silicate, calcium aluminate, calcium oxide, base resin, calcium tungstate, and titanium oxide

### Determination of pH

Freshly extracted single-rooted teeth were sectioned longitudinally. The sectioned root was place at the bottom of a sterile 12-well plate (Star Lab, Milton Keynes, UK). 0.5 g of each medicament (*n* = 3) was placed over the sectioned tooth in the root canal and 10 ml of Hank’s Balanced Salt Solution (HBSS, Gibco®, Grand Island, NY, USA) was dispensed. The plates were then stored at 37 °C and 5 ml of the HBSS solution was collected at specified time intervals of 30, 60, and 120 min and 1, 3, 7, 14, 21, and 28 days. The pH was measured at room temperature using a pH meter (FiveEasy Cond Meter F30, Mettler Toledo, Greifensee, Switzerland) and a temperature-compensated pH electrode (pH electrode InLab Easy, Mettler Toledo, Greifensee, Switzerland) previously calibrated at five points (pH 2.00, pH 4.01, pH 7.00, pH 9.21, pH 12.00) using buffer solutions (Technical Buffer Solutions, Mettler Toledo, Greifensee, Switzerland). The HBSS solution was then returned to the original 12-well plate and stored back at the temperature of 37 °C. Simultaneously, a parallel experiment was conducted with 0.5 g of each medicament (*n* = 3) placed at the bottom of the well of sterile 12-well plate (Star Lab, Milton Keynes, UK). Furthermore, a negative control group was incorporated, consisting of 10 ml of HBSS added without any medicament.

### Direct contact test

To assess the antimicrobial activity of each medicament, direct contact testing was performed against planktonic suspensions of four microbial strains including *E. faecalis*, *S. mitis/oralis*, *C. acnes*, and *S. Epidermidis*. On fastidious anaerobic agar (FAA, Thermo ScientificTM, Loughborough, UK) supplemented with 5% horse blood, all bacteria were grown anaerobically for seven days at 37 ◦C in an anaerobic workstation (MACS-MG-1000, Don Whitley Scientific Ltd., Bingley, UK).

One aliquot of the bacterial culture was inoculated into 3 mL of brain heart infusion (BHI, Lab M, Bury, UK) broth and the absorbance was adjusted with BHI to 0.5 at 550-nm optical density to obtain bacterial concentrations of 3 × 10 mL, using a Labsystem iEMS Reader (MF, Basingstoke, UK).

For each microbial species (1–4), a separate 24-well flat bottom plate (Star Lab, Milton Keynes, UK) was used; 200 µL of each medicament was injected in the base of individual well (*n* = 24, each medicament group was assigned to 4 bacteria used in this study, with the experiment performed in triplicates). Next, 500 µL of bacterial suspension was added on the top of each medicament, without further mixing. This approach simulates a clinical scenario where the medicament and bacteria are not actively mixed but remain in direct contact. In this way, the interaction between the surface of a medicament and the bacteria occurs passively, reflecting real conditions more accurately.

A bacterial suspension with no medicament (*n* = 3) was also added and used as the positive control.

To ensure the absence of any cross-contamination in culture medium, sterilised BHI was injected into empty wells, which assigned as a negative control group and helped in reducing the evaporation of the main culture media.

The 24-well plates were incubated anaerobically at 37 ◦C. The quantitative viable counts were determined by performing serial dilution at different time intervals including 1 and 48 h as well as 7, 14, 21 and 28 days after direct contact between the microbial suspension and medicament; 100-µL aliquots were plated onto triplicated FAA plates. Following seven days of incubation of the plates at 37 °C, the CFU mL-1 count and the logarithmic transformation (log 10) were determined.

### XTT assay

A colorimetric 2,3-Bis-(2-Methoxy-4-Nitro-5-Sulfophenyl)−2H-Tetrazolium-5-Carboxanilide (XTT) assay was performed to evaluate the metabolic activity and number of viable bacterial cells after direct contact with the medicaments at the same time intervals of the direct contact test. At each time point, the XTT cell proliferation kit II (Roche, Mannheim, Germany) was firstly defrosted at room temperature for 20 min and solution of 5 ml XTT labelling reagent and 0.1 ml of electron coupling agent was then prepared following the manufacturer’s instructions. Next, 100 µl aliquots of bacterial suspensions from BC, CH and positive control wells (*n* = 3 of each group) were transferred to 48-well plates (Star Lab, Milton Keynes, UK), mixed with 100 μl of the freshly prepared XTT solution, and incubated anaerobically for 4 h at 37 °C. After incubation, the content from each well was transferred to 96-well plates in duplicates (2 × 100 μl) to measure absorbance at the wavelengths of 450 nm and 689 nm using the microplate reader (Multiscan FC 357, Fisher Scientific, UK). The results were corrected with a blank, and the values averaged for the test and control groups.

### Particle characterisation and surface area analysis

We examined freshly mixed BC and CH pastes using Keyence VHX-7000 4 K digital microscope (Keyence, Osaka, Japan). Each medicament was dispensed directly onto a microscopic glass slide and inspected using a combination of full-ring and coaxial lighting, along with a high-magnification objective lens (VHX-E500; magnification × 2500). Particle size was quantitatively assessed using a measurement tool within the digital microscope.

Additionally, BC and CH powders were analysed using a high-

resolution JEOL JSM 7800 F Prime scanning electron microscope (SEM) (JEOL UK Ltd, Welwyn Garden City, UK). To prepare the powder, 0.1 g of BC and CH pastes were placed into eppendorf tubes, followed by the addition of 1 ml of ethanol (Ethyl alcohol, ≥ 100%, Sigma Aldrich, Dorset, UK). Tubes were microcentrifuged for 15 s at 15.0 xg (Heraus Fresco 21, Thermo Scientific, Loughborough, UK), followed by the removal of supernatant. The process was repeated three times. Afterwards, ethanol was added again and left to evaporate over a period of 72 h at room temperature. Prior to SEM analysis, samples were coated with 8 nm-thick conductive gold coating to enhance imaging quality. Because of the irregular shapes of the particles, surface areas of randomly selected particles (*n* = 30) were analyzed using ImageJ software (National Institutes of Health, Bethesda, Maryland, USA), and the average sizes of both BC and CH were calculated.

### Statistical analysis

Statistical analysis was performed using a two-way analysis of variance (ANOVA), followed by post hoc Tukey’s and Šídák’s tests (GraphPad, Prism, La Jolla, USA). The significance threshold and confidence level were set at p ≤ 0.05 and 95% confidence interval, respectively. Quantitative data were represented graphically as mean values with error bars indicating standard deviations, wherever applicable.

## Results

### pH variation of the endodontic medicaments

**Fig. 1:**
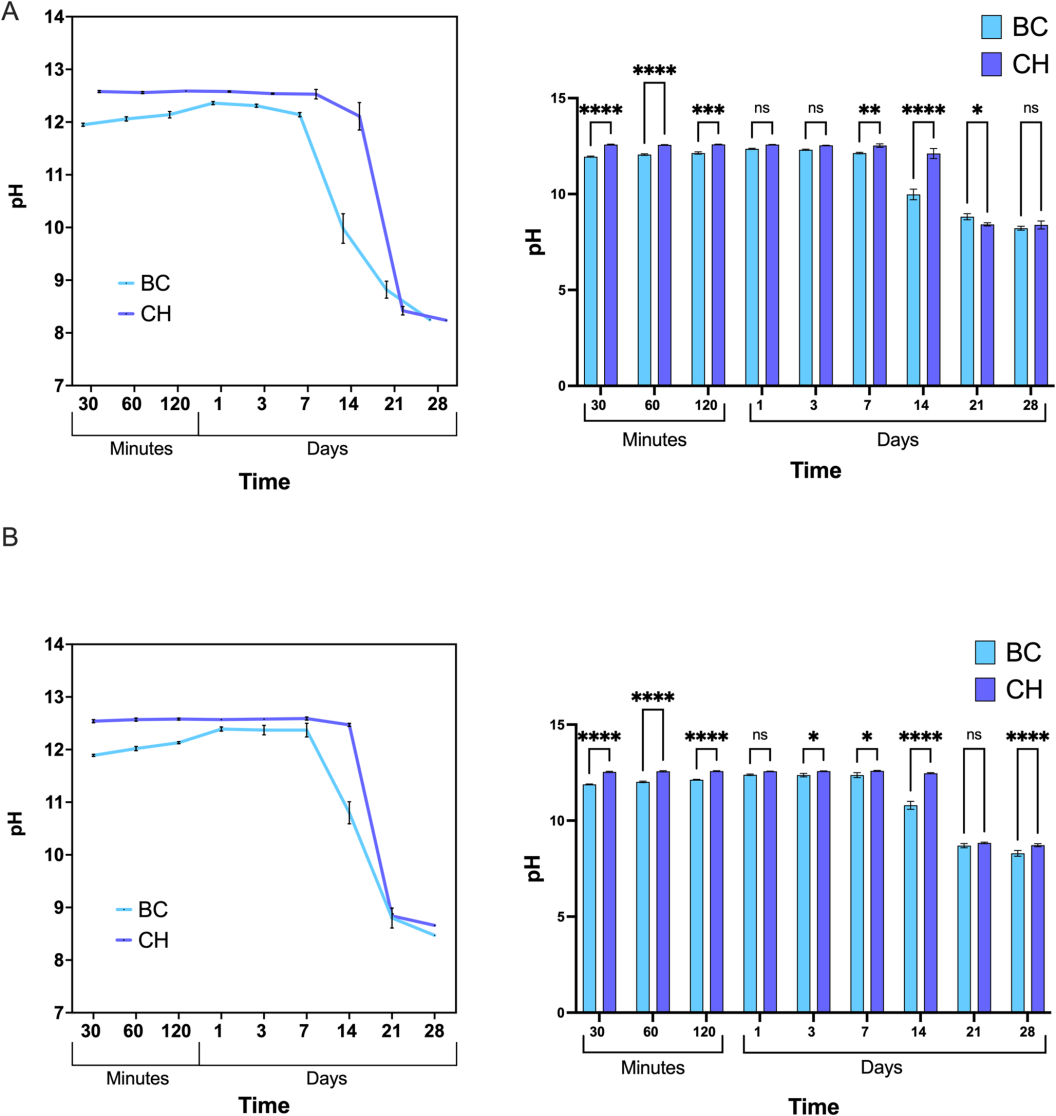
pH variation of endodontic medicaments. (**A**) The impact of BC and CH intracanal medications on pH changes in HBBS over a 28-day timeframe, with a sectioned tooth root. The accompanying bar graph illustrates significant differences at indicated time intervals. The results are presented as the mean ± standard deviation (with error bars) of pH values tested after 30, 60, and 120 min, as well as 1, 3, 7, 14, 21, and 28 days. (**B**) Linear graphs illustrating the pH values of intracanal medicaments without the root throughout the 28-day observation period. Bar graphs indicate the statistical significance between CH and BC medicaments in medium without the root.

The pH changes of the HBSS solution in contact with the medicaments only showed that CH maintained significantly higher pH values than BC over 120 min (*p* < 0.0001) and maintained a greater pH across all time points until day 14 (Fig. 1). In contrast, BC exhibited a gradual increase in pH up to day 1, followed by a plateau up to day 7 when it reached a maximum pH of 12.59 ± 0.03. The pH of the HBBS in presence of BC exhibited a gradual decrease in pH over the experimental period reaching a value of 8.47 at day 28 whilst CH maintained a higher pH value when compared to BC (Fig. 1A). Both medicaments, CH and BC showed a reduction in pH over the study period however BC showed a decrease in pH from 12.47 ± 0.03 (day 14) to 8.66 (day 28), whilst CH maintained significantly higher pH compared to BC (*p* < 0.0001).

The pH variation recorded for both CH and BC medicaments in presence of the root dentine showed differences in pH at different time intervals, however there were no statistical differences at 28 days (*p* = 0.65). The presence of root dentin did not exhibit an effect on pH and the consistently higher pH of CH was found to persist.

### Direct contact tests

#### Effect of medicaments on streptococcus mitis/oralis

**Fig. 2:**
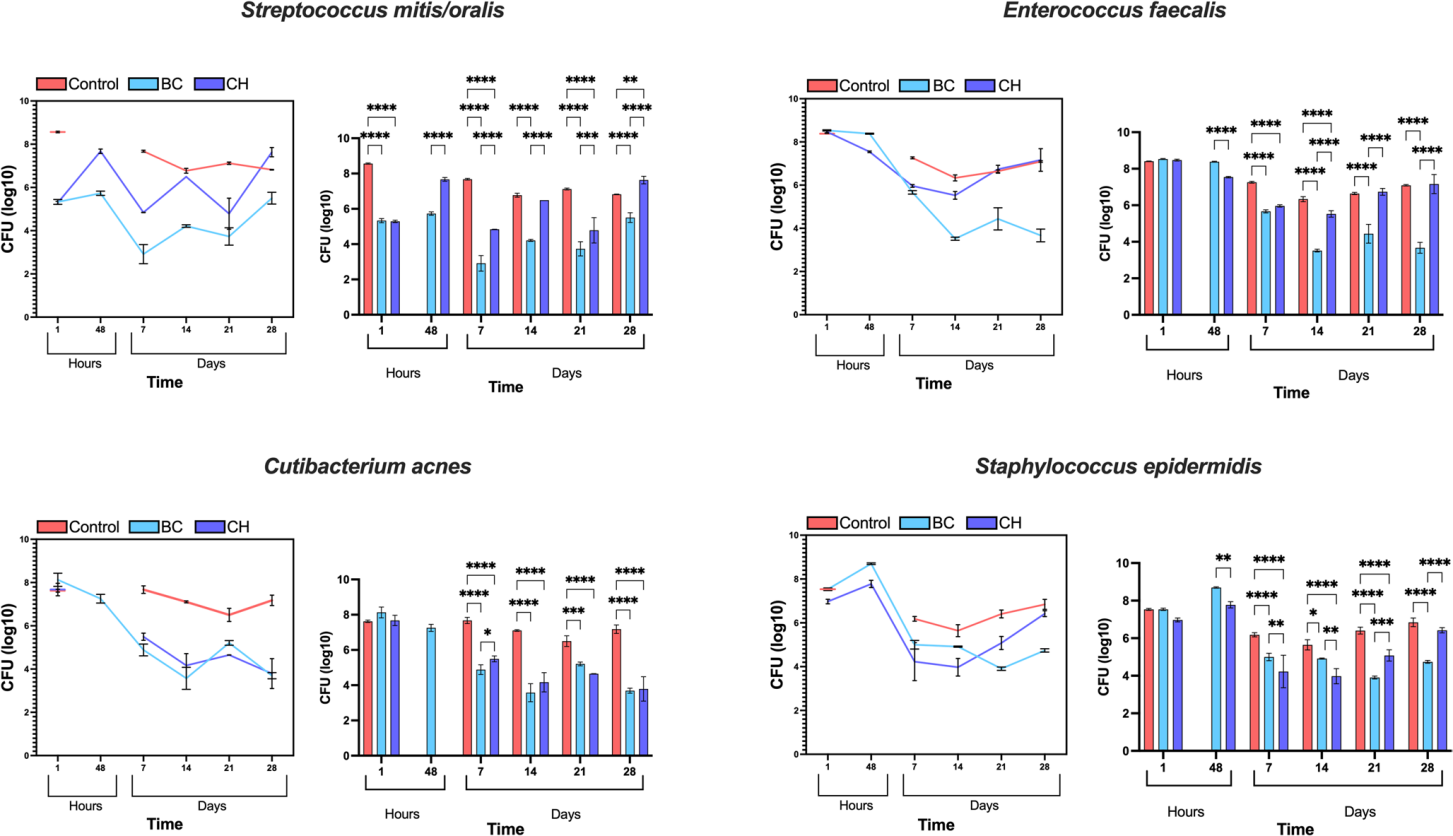
Quantitative viable counts of Streptococcus mitis/oralis, Enterococcus faecalis, Cutibacterium acnes, and Streptococcus epidermidis following direct contact test, comparing the effectiveness of each intracanal medicament, BC (blue) and CH (purple) at 1, 48 h and 7, 14, 21, 28 days. Red lines/columns represent the control for each bacterium. The data are expressed as log10 (CFU per sample ml^*−1*^), representing the mean number of bacteria and standard deviation (error bars). The significance level is denoted as follows: * *p* ≤ 0.05, **p < 0.01, ***p < 0.001, ****p < 0.0001

The direct contact tests using S. mitis/oralis exhibited similar viable cell counts for BC (5.33 ± 0.11) and CH (5.29 ± 0.07) at 1 h incubation which subsequently increased after 48 h to 5.73 ± 0.1 for BC and 7.67 ± 0.1 for CH. BC and CH presented the lowest value of viable counts of 2.91 ± 0.44 and 4.84 ± 0.01, respectively, at 7 days with BC being significantly more effective than CH (*p* < 0.0001) (Fig. 2). At 14 days, both medicaments exhibited a rise in viable counts. By day 21, BC (3.73 ± 0.4) and CH (4.79 ± 0.71) experienced another decline in the number of viable cells, followed by a sharp rise at 28 days, with BC (5.50 ± 0.3) returning to similar levels as observed at 1 h, while CH (7.63 ± 0.21) showed a marked increase in comparison. In summary, BC demonstrated a more pronounced reduction in viable cell counts showing higher antibacterial effect over the observed period.

#### Effect of medicaments on Enterococcus faecalis

Initially, all medicaments exhibited equivalent viable cell counts at 1 h. However, after 48 h, only CH (7.54 ± 0.04) showed a slight decrease in viable cells (Fig. 2). By day 7, there was a comparable reduction in viable cell counts with BC measuring 5.66 ± 0.08 and CH at 5.96 ± 0.06. At 14 days, BC (3.52 ± 0.07) demonstrated significantly greater antimicrobial effect compared to CH (5.52 ± 0.18) (*p* < 0.0001). This difference persisted at 21 days, with BC (4.43 ± 0.51) and CH (6.73 ± 0.18) (*p* < 0.0001). Finally, at 28 days, BC (3.67 ± 0.3) maintained significant antimicrobial effectiveness compared to CH (7.16 ± 0.52) (*p* < 0.0001), with CH showing no difference from the control group. In summary, while CH initiated the reduction in viable cell counts, BC exhibited a consistent and significant decrease overall.

#### Effect of medicaments on Cutibacterium acnes

At 1 h, BC (8.12 ± 0.3) exhibited higher cell counts compared to CH (7.67 ± 0.3) (Fig. 2). By 48 h, only BC data was available, indicating a reduction in viable cell counts. The reduction persisted from day 7 to 14, with BC reaching its Lowest detectable value at 14 days (3.57 ± 0.5). At day 21, both BC (5.21 ± 0.1) and CH (4.65 ± 0.005) showed an increase in viable cell counts but persisted significantly lower than the positive control group (*p* = 0.0002 and *p* < 0.0001, respectively). By 28 days, both BC (3.69 ± 0.14) and CH (3.78 ± 0.7) experienced a decline in cell counts. It can be concluded that both medicaments demonstrated similar effectiveness against C. acnes.

#### Effect of medicaments on Staphylococcus epidermidis

After 1 h, BC exhibited higher viable cell counts (7.53 ± 0.06) compared to CH (6.97 ± 0.1) (Fig. 2). An increase in viable cell counts was noted for BC (8.69 ± 0.04) and CH (7.77 ± 0.17) at 48 h. After 7 days, a significant decline in viable cell counts was noted for CH (4.23 ± 0.86) when compared to BC (5.00 ± 0.2) (*p* < 0.01). At 14 days, a plateau in viable cell counts was seen for both BC (4.91 ± 0.03) and CH (3.97 ± 0.4) whilst at 21 days, BC (3.9 ± 0.07) exhibited significantly more microbial killing than CH (5.07 ± 0.3) (*p* < 0.001). An increase in viable cell counts at the final measurement between BC (4.74 ± 0.08) and CH (6.42 ± 0.14) presented with a significant difference between the two medicaments (*p* < 0.0001), as well as BC and the positive control group (*p* < 0.0001). In conclusion, while both medicaments maintained similar viable cell counts over the observed time, the resulting viable cell counts for BC were distinctly lower at the final time point.

### XTT Assay

#### Effect of medicaments on Streptococcus mitis/oralis

**Fig. 3:**
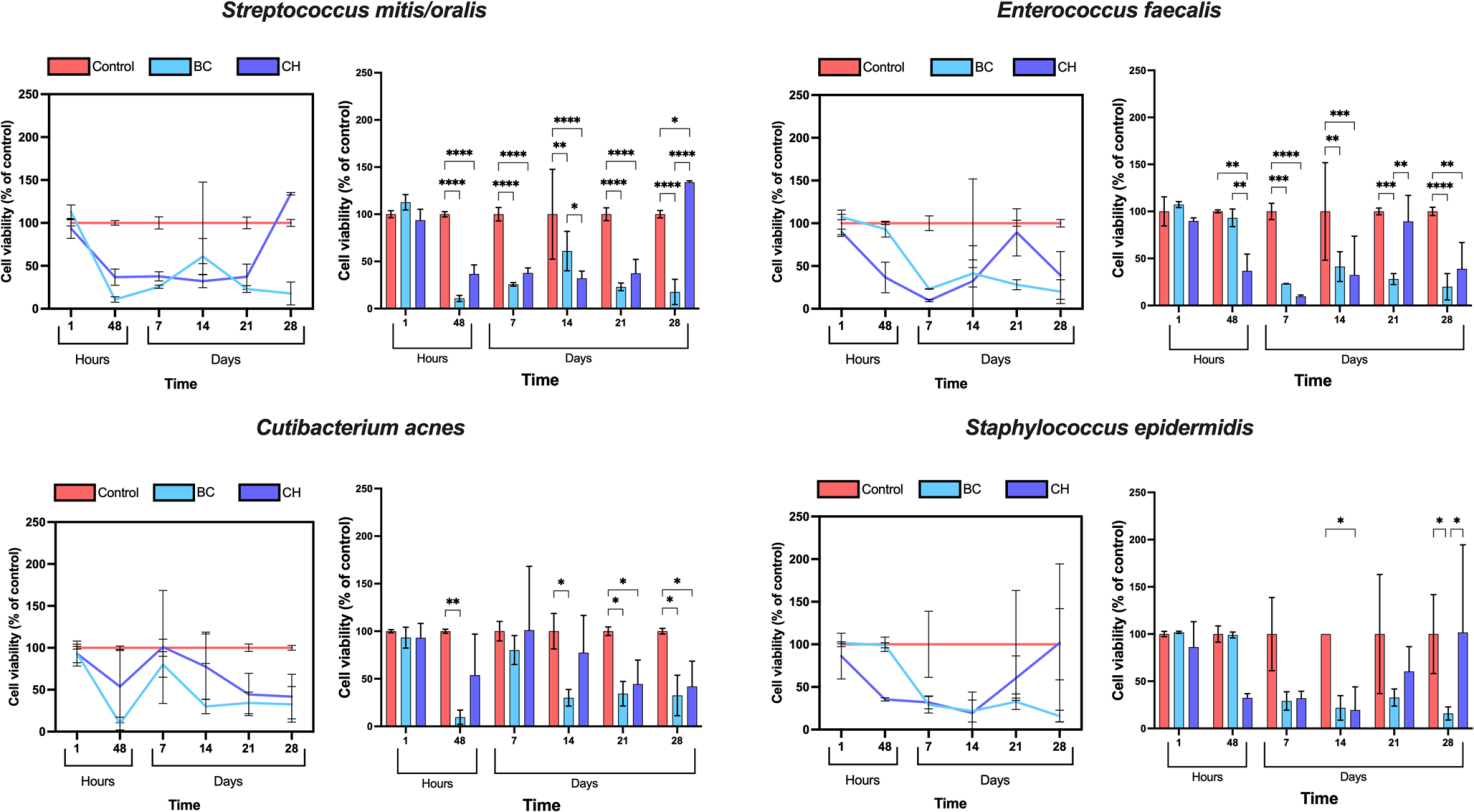
XTT assay demonstrates the percentage of viable cells as percentage of the control for *Streptococcus mitis/oralis, Enterococcus faecalis, Cutibacterium acnes, and Streptococcus epidermidis*. Red lines/columns represent the control for each bacterium. The results are reported as the mean percentage of viable bacteria ± standard deviation (error bars). The significance level is denoted as follows: * *p* ≤ 0.05, **p < 0.01, ***p < 0.001, ****p < 0.0001

At 1 h, BC recorded higher number of viable cells than the control group whilst CH presented just below the positive control (Fig. 3). Both CH and BC demonstrated a significantly rapid reduction (*p* < 0.0001) in cell viability compared to the controls after 48 h, resulting in 36.72 ± 9.55% and 10.70 ± 3.22% viable cells, respectively. On days 7, 14, and 21, the effect of CH remained consistent, followed by a rapid increase on day 28, resulting in 133.97 ± 1.46% viable cells. In contrast, BC peaked earlier with a gradual increase from 48 h up to 14 days (from 10.7 ± 3.22 to 61.02 ± 20.95%), and subsequently started declining until reaching a significantly lower (*p* < 0.0001) percentage (17.75 ± 13.41%) of viable cells compared to CH and control at the end of the experiment. In summary, BC showed a more distinct reduction in cell viability over the 28 days compared to CH.

#### Effect of medicaments on Enterococcus faecalis

From 1 h to the seventh day, both CH and BC demonstrated a similar trend, with CH reducing the number of viable cells from 89.78 ± 3.37% to 9.67 ± 1.45%, and BC from 107.23 ± 3.19% to 23.13 ± 0.26% (Fig. 3). Except for the minor peak (41.32 ± 15.82) that was observed in the BC treatment at day 14, the declining trend continued up to day 28 (19.97 ± 13.97%). In contrast, the number of viable cells increased to 89.35 ± 27.7% when treated with CH after 21 days and followed by a steep decline at the final measurement (day 28). Despite the notable decline, the number of viable cells remained higher when treated with CH (38.99 ± 27.9%) compared to the BC-treated groups at the end of the experiment. Although CH showed a significant reduction (*p* < 0.0001) up to day 7, BC treatment resulted in an overall improved reduction in cell viability.

#### Effect of medicaments on Cutibacterium acnes

After 1 h, there were no significant differences in cell viability between treated groups and positive control (Fig. 3). BC showed a significant (*p* = 0.0022) reduction in viable cells up to 48 h (9.64 ± 7.5%) compared to the control. Subsequently, both BC and CH treatments resulted in an increased cell viability up to day 7, with BC at 80.17 ± 15.2%, and CH at 101.04 ± 67.32% of viable cells. Afterwards, both BC and CH medicaments exhibited a significant (BC *p* = 0.024, and CH *p* = 0.03) decrease in cell viability until the final day compared to control, thereby recording the measurements of 32.5 ± 21.3% and 41.9 ± 26.56% viable cells, respectively. Consequently, it could be concluded that both medicaments demonstrated similar effect against C. acnes.

#### Effect of medicaments on Staphylococcus epidermidis

CH reduced the number of viable cells to 19.51 ± 24.55% within 48 h, and ultimately resulted in high cell viability at day 28 (101.7 ± 92.73%) (Fig. 3). In contrast, BC did not show any reduction in cell viability within the first 48 h. However, a sharp decline was observed at day 7 (29.12 ± 9.74%) which continued decreasing steadily until the final day, resulting in a significantly lower (*p* = 0.029) number of viable cells (15.76 ± 6.93%) than the above-mentioned CH. In conclusion, although CH initiated the reduction in viable cells at an earlier time point, BC medicament showed an increased overall reduction in cell viability over a designated period.

### Particle morphology and surface area

**Fig. 4:**
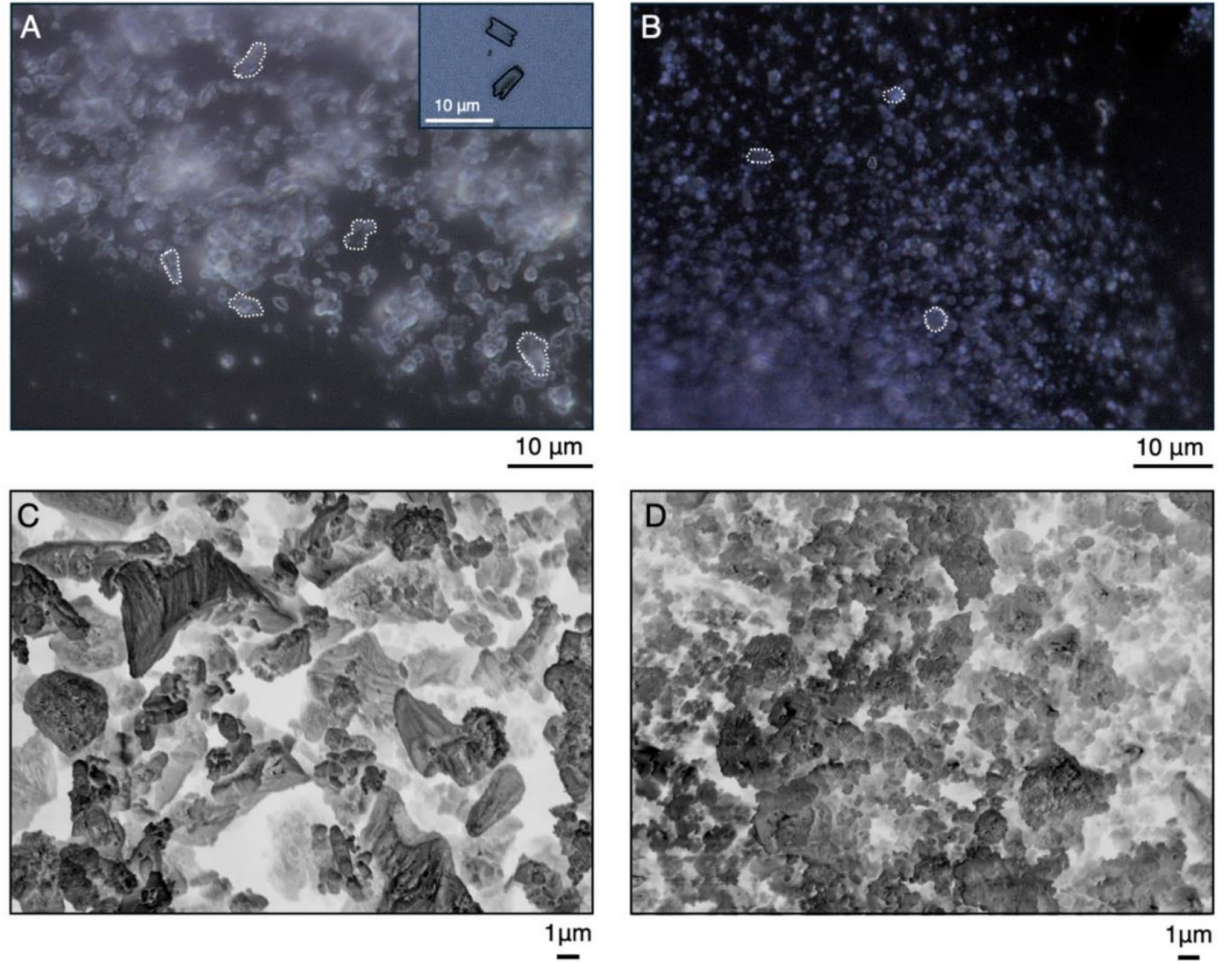
Digital microscope (**A** and **B**, magnification × 2500) and SEM images (**C** and **D**, magnification × 4000 and × 5500, respectively) depict the morphology of intracanal medicaments. Rod-shaped particles and triangles (**A** and **C**) of variable surface areas ranging from 1.62 μm^2^ to 33 μm^2^ were observed in CH medicament. BC was composed of numerous rounded particles at both nano- and micro-scales, with surface area ranging from 0.21 μm^2^ to 2.93 μm.^2^ (**B** and **D**)

Inspection of intracanal medicaments under digital and SEM microscopes revealed the presence of dimensionally and morphologically different particles. Accordingly, CH powder exhibited irregular rod-and triangle-like shape, with an average particle surface area of 8.15 ± 7.6 μm^2^, ranging from a minimum of 1.62 μm^2^ to a maximum of 33 μm^2^ (Fig. 4A and C). In contrast, BC consisted of numerous grouped and individual spherical particles, with surface areas ranging from a minimum of 0.21 μm2 to a maximum of 2.93 μm2, and the average surface area of 0.88 ± 0.7 μm^2^ (Fig. 4B and D).

## Discussion

Effective removal of bacterial biofilm during endodontic treatment is essential to prevent the subsequent reinfection. The objective of this research was to assess the impact of a calcium silicate-based bioceramic medicament (BC Temp) on the survival of four common endodontic bacterial strains found in both primary and secondary root canal infections: *S. mitis/oralis*, *E. faecalis*, *C. acnes*, and *S. epidermidis* [13–15], over a 28-day period. A comparison was made with a calcium hydroxide medicament.

The role of alkalinity is well-established feature of the antimicrobial effect of intracanal medicaments. pH values of 12 and above not only employ antimicrobial action but can also neutralise bacterial by-products, create a favourable environment for tissue repair, and induce hard tissue regeneration [16]. To achieve these effects, the pH values need to remain stable over prolonged period, ideally for the time that medicaments are designated to be inside the canal. The recommended duration for an intracanal medicament to exert its full effect is 2 to 4 weeks, although this can vary from 1 week to several months [17]. Considering clinical application, we examined the CH and BC efficacy over extended period of time – 28 days. Our results indicated that both CH and BC demonstrated high alkaline activity during the first 7 days, consistent with findings from other studies [18]. While similar results were observed up to day 7, we showed significant changes in behaviour from this time point onwards. Specifically, BC exhibited faster pH decline, starting from day 7, while CH maintained its alkalinity until the day 14. This is also in agreement with previous findings which showed that due to the low solubility in water, CH can preserve the high pH values over an extended time [19, 20]. On the other hand, manufacturer suggests that BC’s high levels of calcium silicates and calcium oxide dissociate when in contact with tissues, forming Ca(OH)_2_, and inducing similar pH values to CH. However, no concrete data is provided on the BC’s ability to preserve high pH value over a long period.

In the present study, both BC and CH demonstrated significant antimicrobial activity against the four planktonic bacteria analysed in the DCT and XTT assay. DCT showed that the CH reduced the CFU for *C. acnes* and *E. faecalis* in the first 48 h. Apart from *C.acnes* and *E.faecalis*, XTT assay results showed reduction in cell viability for other bacteria types treated with CH after 48 h. BC treatment exhibited a delayed effect on selected bacterial species. According to a DCT test, a reduction in CFU for *S. epidermidis*, *E. faecalis and C. acnes* was observed after 7 days of treatment with BC. XTT assay revealed BC’s prompt reduction in viable cells for *S. mitis/oralis* and *C. acnes* within 48 h. *S. epidermidis* and *E. faecalis*, however, only responded after 7 days of treatment with BC. Despite this delayed effect for some bacterial species, BC consistently exhibited a Lower and more stable impact on bacterial viability than CH after 14 days. Nevertheless, it is important to interpret XTT findings with caution due to the limitations of the assay, given the susceptibility of the CH and BC to dissolve into the surrounding medium. Specifically, we noticed fluctuating XTT results, with occasional shifts towards higher absorbance readings for both medicaments during the 28-day duration. We attributed these variations to the gradual dissipation of particles which resulted in elevated readouts. Existing studies on this topic also highlight the potential interference of XTT by small-scale particles [21–23]. Consequently, the dispersion of micro- and nanoparticles following material setting represented a limitation of our study towards designing an experiment with mature biofilms on BC and CH medicaments. In such a scenario, the required medium refreshment during biofilm growth, combined with fluid shear forces, would likely lead to early biofilm disruption and detachment. This could result in false-negative outcomes, reduced bacterial attachment, lower CFU counts, and increased variability between replicates. In clinical practice, intracanal medicaments are typically applied after extensive instrumentation and irrigation, procedures that significantly disrupt the biofilm architecture and lead to the potential release of microbial cells into a planktonic state. Hence, in line with previous studies on this topic, we selected planktonic bacterial models and the DCT [18, 24–26] as our primary approach due to its suitability for direct quantification of CFU. Meanwhile, the XTT assay results serve as a valuable complementary indicator of metabolic activity that should be interpreted alongside CFU counts, particularly when testing particle-releasing medicaments. However, our XTT results are consistent with previous studies which found that calcium silicate products exhibited pronounced antimicrobial efficacy against bacteria in a planktonic medium [27]. Carvalho et al. reported that the CH exhibited abundant levels of Ca^2+^ and OH^−^ release upon the application [28]. High yields of fast-releasing Ca^2+^ ions are responsible for cell stimulation, proliferation and migration, whilst the release of OH^−^ ions facilitates the establishment of an alkaline environment [29]. It is plausible that the Ca^2+^ and OH^−^ ions synergy increases the antimicrobial activity swiftly after the application of medicament yielding a rapid effect on all bacteria types in this study as demonstrated in the CFU and XTT assays.

Among the bacteria linked to primary and secondary endodontic lesions, *E. faecalis* is notably the most resistant and withstands the high pH environment of CH medicament [7, 30]. *E.faecalis* can survive in the depths of dentinal tubules for long periods by adhering to collagen and forming biofilms [31]. Therefore, medications must be effective long enough to maximise the eradication of *E. faecalis* [32]. As mentioned previously in the text, DCT revealed that BC medicament achieved a more sustained reduction in *E. faecalis* colonies than CH, despite CH’s rapid effectiveness against other bacterial species. It was demonstrated that both BC and CH medicaments increase the pH value of the environment, but due to *E.foecalis* resistance, it is plausible that pH alone is not the only factor impacting the bacterial survival. Therefore, we suspected that the higher efficacy of BC towards *E.faecalis* could be due to its nano-scale particle size compared to CH [33]. Our measurements showed that BC’s particles have smaller surface area compared to CH. Broadly speaking, particle volume is an important factor in membrane translocation [34]. For example, larger particle surface area of CH increases its maximum possible contact area with the membrane, making membrane translocation more difficult. Conversely, smaller nanoparticles, like those in BC, have a greater surface area to volume ratio which enhances their ability to contact and penetrate bacterial membranes [34–36]. Literature also suggests that nano-sized particles have demonstrated a broad spectrum of antimicrobial properties, with greater activity against Gram-positive bacteria [37]. All bacteria tested in this study were Gram-positive, characterised by a thin peptidoglycan layer, teichoic acid, and numerous pores that facilitate the penetration of external molecules. This results in damage and disruption of the cell membrane, leading to cell death by loss of cytoplasmic contents [35, 37]. Other mechanisms for BC nanoparticles' antimicrobial effects may include generating reactive oxygen species (ROS), inducing intracellular antimicrobial effects, interacting with DNA and proteins, or the negative charge of Gram-positive bacterial membranes that potentially attracts NPs and enhances penetration [37]. Furthermore, due to their size, NPs can mimic large protein complexes, disrupting metabolic pathways and inhibiting bacterial growth, but further investigation is needed to fully understand the inhibition mechanisms of BC nanoparticles [36]. The impact of nanoparticle shape on membrane translocation and antimicrobial effects is still not well understood. Nonetheless, Yang et al. [34] demonstrated that altering the shape of nanoparticles while keeping their volume constant, and *vice versa*, can affect their penetration rate through the cellular membrane. They concluded that penetration is influenced by the contact area between the particle and the membrane; hence, the spherical and quasi-spherical shapes of BC, as opposed to the rod and triangular forms of CH, could explain the higher antimicrobial efficacy of particles of the same scale. Further supporting the efficacy of nano-sized particles, Dianat et al. [38] conducted an in-vitro study and found improved efficacy of nano-particle CH at dentinal depths of 200 µm and 400 µm compared to the conventional particle size of CH. Apart from geometry considerations, the ingredients of medicaments can also influence the antibacterial effects of BC. For example, the presence of titanium dioxide within the formulation of BC Temp could potentially contribute to the antibacterial properties. The incorporation of TiO_2_ nanoparticles into many biomaterials such as composites has shown sustained release rates with the ability to reduce bacterial colonisation [39]. This may be explained by the physicochemical and biological activities of these particles in addition to their unique photocatalytic action.

## Conclusion

In summary, planktonic microorganisms were susceptible to the antibacterial action of freshly generated endodontic medicaments. The study also showed that a calcium silicate-based medicament possessed antimicrobial properties comparable to or potentially better than conventional calcium hydroxide medicament. While maintaining a pH similar to calcium hydroxide, which has a well-established antibacterial effect, the calcium silicate medicament may achieve a broader bactericidal effect due to its smaller particle size. This may enable BC to act on bacteria both directly, by more readily penetrating the membranes; and indirectly, by securing the alkaline environment.

## Data Availability

No datasets were generated or analysed during the current study.
